# Distinct Functions for the *Drosophila* piRNA Pathway in Genome Maintenance and Telomere Protection

**DOI:** 10.1371/journal.pgen.1001246

**Published:** 2010-12-16

**Authors:** Jaspreet S. Khurana, Jia Xu, Zhiping Weng, William E. Theurkauf

**Affiliations:** 1 Program in Cell and Developmental Dynamics and Program in Molecular Medicine, University of Massachusetts Medical School, Worcester, Massachusetts, United States of America; 2 Department of Biomedical Engineering, Boston University, Boston, Massachusetts, United States of America; 3 Program in Bioinformatics and Integrative Biology and Department in Biochemistry and Molecular Pharmacology, University of Massachusetts Medical School, Worcester, Massachusetts, United States of America; Stowers Institute for Medical Research, United States of America

## Abstract

Transposons and other selfish DNA elements can be found in all phyla, and mobilization of these elements can compromise genome integrity. The piRNA (PIWI-interacting RNA) pathway silences transposons in the germline, but it is unclear if this pathway has additional functions during development. Here we show that mutations in the *Drosophila* piRNA pathway genes, *armi*, *aub*, *ago3*, and *rhi*, lead to extensive fragmentation of the zygotic genome during the cleavage stage of embryonic divisions. Additionally, *aub* and *armi* show defects in telomere resolution during meiosis and the cleavage divisions; and mutations in *lig-IV*, which disrupt non-homologous end joining, suppress these fusions. By contrast, *lig-IV* mutations enhance chromosome fragmentation. Chromatin immunoprecipitation studies show that *aub* and *armi* mutations disrupt telomere binding of HOAP, which is a component of the telomere protection complex, and reduce expression of a subpopulation of 19- to 22-nt telomere-specific piRNAs. Mutations in *rhi* and *ago3*, by contrast, do not block HOAP binding or production of these piRNAs. These findings uncover genetically separable functions for the *Drosophila* piRNA pathway. The *aub*, *armi*, *rhi*, and *ago3* genes silence transposons and maintain chromosome integrity during cleavage-stage embryonic divisions. However, the *aub* and *armi* genes have an additional function in assembly of the telomere protection complex.

## Author Summary

Transposons and other selfish genetic elements make up a significant fraction of all eukaryotic genomes, and the piRNA pathway appears to have a conserved function in transposon silencing and genome maintenance. However, other functions for this pathway have not been fully explored. Telomeres must be protected from recognition as DNA breaks by the repair machinery, which can covalently ligate unprotected chromosome ends and thus disrupt meiotic and mitotic chromosome segregation. We show that mutations in a subset of piRNA pathway genes disrupt meiotic and mitotic chromosome separation and that these segregation defects are suppressed by a mutation that blocks ligation of non-homologous DNA ends. These mutations also disrupt assembly of the telomere protection complex and reduce expression of a subpopulation of 19- to 22-nt telomere-specific RNA. We therefore propose that a subpopulation of short piRNAs direct assembly of the telomere protection complex.

## Introduction

*Drosophila* piRNAs have been implicated in transposon silencing and maintenance of genome integrity during female germline development. However, piRNA pathway mutations lead to complex developmental phenotypes [1], [2], [3], [4], and piRNAs have been implicated in control of gene expression [5], [6], [7], [8]. Furthermore, the majority of piRNAs in other systems, including mouse testes, are not derived from repeated elements [9], [10], [11], [12], [13]. The full extent of piRNA functions thus remains to be explored.

Mutations in the majority of *Drosophila* piRNA pathway genes disrupt asymmetric localization of RNAs along the axes of the oocyte, and lead to maternal effect embryonic lethality [1], [2], [3], [4]. The axis specification defects linked to several of piRNA pathway mutations are dramatically suppressed by a null mutation in *mnk*, which encodes a Checkpoint kinase 2 (Chk2) homolog required for DNA damage signaling, indicating that the loss of asymmetric RNA localization is downstream of DNA damage [1], [2]. Oocyte patterning defects generally lead to embryonic lethality, but the *mnk* allele that suppresses the axis specification defects associated with piRNA mutations does not suppress embryonic lethality [1], [2], [3]. piRNAs thus have an essential function during embryogenesis that is independent of Chk2 activation and DNA damage signaling. To gain insight into potential new functions for the piRNA pathway, we have characterized the embryonic lethality associated with four piRNA pathway mutations. These studies reveal a novel function for a subset of piRNA genes in assembly of the telomere protection complex, and suggest that this process is directed by a subpopulation of 19–22 nt piRNAs.

## Results/Discussion

The *armi* and *aub* genes encode a putative RNA helicase and a piRNA binding PIWI Argonaute protein, and recent studies suggest that they have distinct functions in piRNA biogenesis [2], [8], [14], [15] Mutations in *aub* dramatically reduce piRNA species that overlap by 10 nt, which is characteristic of ping-pong amplification, while *armi* mutations reduce total piRNA production but enhance the ping-pong signature [15]. Mutations in *aub* and *armi* lead to maternal-effect embryonic lethality, however, suggesting that these genes share an essential function. To gain insight into the lethality associated with these mutations, we first analyzed DNA break accumulation during oogenesis. Germline-specific DNA breaks normally form during early oogenesis, as meiosis is initiated [16]. In several piRNA mutants, however, DNA breaks persist, which could compromise the female pronucleus and thus lead to genetic instability in the early zygote [2], [14]. DNA breaks trigger phosphorylation of histone H2Av, producing γ-H2Av foci near the break sites [17]. In wild-type ovaries, γ-H2Av foci begin to accumulate in region 2 of the germarium, as meiotic breaks are formed [16]. These foci are significantly reduced in stage 2 egg chambers, which have completed meiotic repair and budded from the germarium. Later in oogenesis, γ-H2Av foci accumulate in the nurse cell nuclei, which undergo endoreduplication. However, these foci remain undetectable in the oocyte [16]. In ovaries mutant for *aub* or *armi*, γ-H2Av foci appear in germarium region 2, but persist in nurse cells and the oocyte through stage 4. By stage 5, however, γ-H2Av foci are undetectable in 50% of *armi* and *aub* mutant oocytes, and are significantly reduced in the remaining oocytes (Figure S1 and data not shown). Both *armi* and *aub* mutations thus increase DNA damage during early oogenesis, but most of the damage in the oocyte appears to be repaired as oogenesis proceeds.

As wild type oocytes mature and initiate meiotic spindle assembly, the major chromosomes form a single mass at the spindle equator and the non-exchange 4^th^ chromosomes move toward the poles [18], [19]. In *OregonR*, we observed distinct 4^th^ chromosomes in 79% of stage 13 oocytes. In stage 13 *aub* and *armi* mutants, by contrast, distinct 4th chromosomes were observed in only 11% and 18% of stage 13 oocytes, respectively (Figure S2, Table S1). However, a single primary mass of chromatin was always observed. These observations are consistent with γ-H2Av data suggesting that DNA breaks formed during early oogenesis are often repaired as the oocyte matures. In addition, both *aub* and *armi* mutations appear to inhibit separation of the small 4^th^ chromosomes, although it is also possible that this small chromosome is fragmented and thus difficult to detect cytologically.

**Figure 1:**
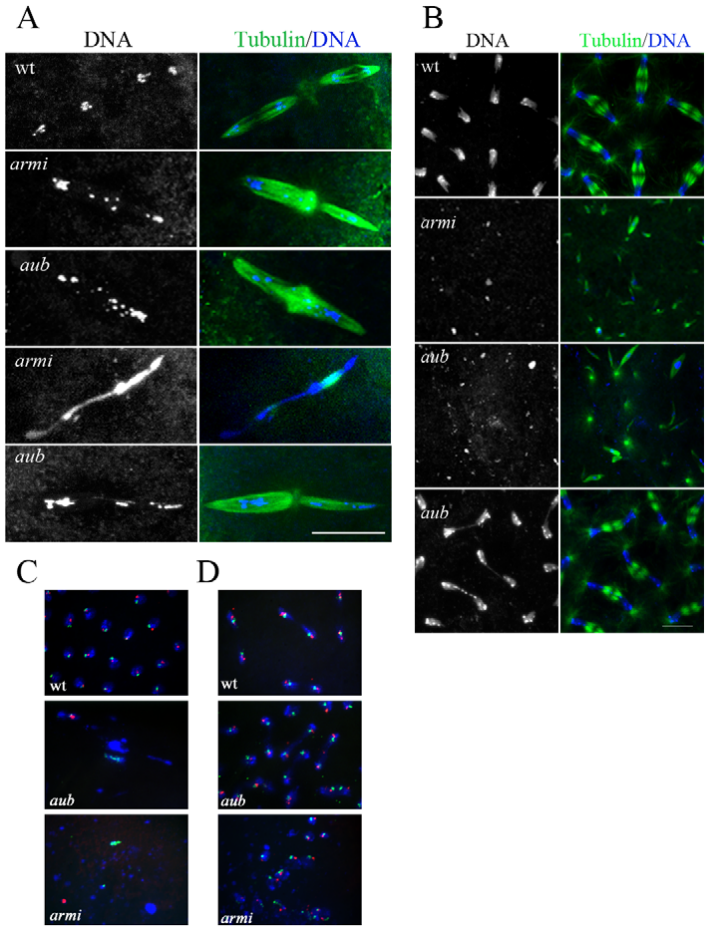
Chromatin organization in piRNA mutant embryos. A. Immunostaining for α-tubulin (green) and DNA (blue) in 0–30-min-old embryos showing chromatin fragmentation and chromatin fusions in *aub* and *armi* mutant embryos during meiosis II. Scale bar is 15 µM. B. Cross-section of 0–3-hr-old embryos during syncytial mitotic divisions showing DNA fragmentation and chromatin bridges during segregation in *aub* and *armi* mutants. Scale bar is 10 µM. C, D. Dual-label FISH for two Y-chromosome-specific satellites, (AATAC)n in green and (AATAAAC)n in red, with DNA in blue showing mis-segregation of these repeats in *aub* and *armi* embryos (C). In contrast, embryos undergoing cleavage mitotic divisions show both the labels in most of the segregating chromatids in *aub* (D).

*Drosophila* oocytes are activated as they pass through the oviduct, which triggers completion of the meiotic divisions. The first meiotic division is completed in the oviduct, but meiosis II can be observed in freshly laid eggs and is characterized by four well-separated meiotic products on tandem spindles (Figure 1A). In *aub* and *armi* mutant embryos, the meiotic chromatin was either stretched across the paired meiotic spindles, or fragmented and spread over both spindles (Figure 1A). No wild type meiotic figures were observed. Breaks thus appear to persist in some stage 14 oocytes, although this does not disrupt the karyosome organization during earlier stages. However, other oocytes appear to have intact chromosomes that fail to resolve during the meiotic divisions.

### Compromised zygotic genomic integrity in piRNA mutants

**Figure 2:**
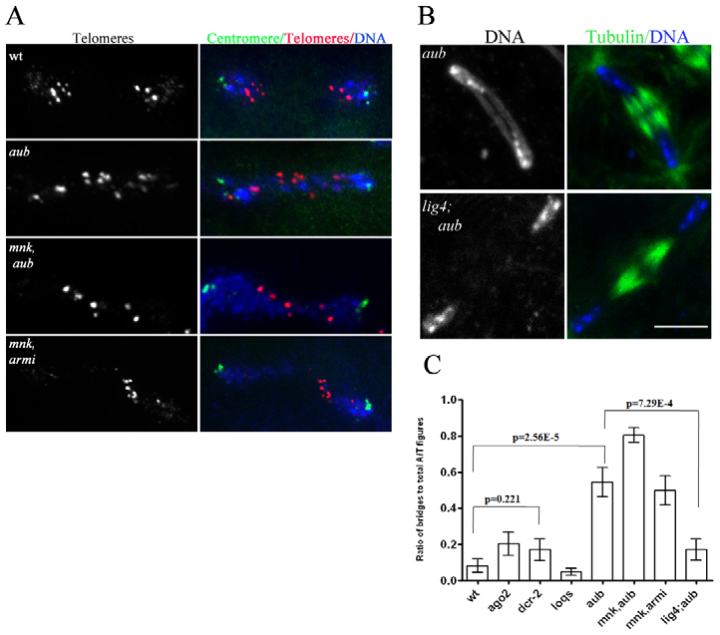
*ligIV*–dependent telomere fusions in piRNA mutants. A. Two-color FISH for a pair of daughter nuclei in anaphase, labeled for centromeric *dodeca* satellite (green) and telomeric transposon, *HeT-A* (red) with DNA (blue) showing telomeres are fused in piRNA mutants. B. Immunostaining for microtubules (green) and DNA (blue) in 0–3 hr-old embryos showing suppression of chromatin bridge formation in *ligIV;aub* embryos. Scale bar is 10 µM. C. Ratio of anaphase/telophase bridges to total anaphase/telophase figures in different genotypes. The data for multiple samples were compared using Anova test, and sample mean was plotted with standard error of mean (SEM) as error bars. A two-tailed t-test was performed for certain pairs and p-values are noted on the graph.

Fertilization and pronuclear fusion initiate 13 rapid cleavage stage mitotic divisions [16]. These divisions are syncytial, but membranes surround the cortical nuclei to form cells following mitosis 13 [20]. 0 to 3-hr old cleavage stage *aub* and *armi* mutant embryos showed two distinct phenotypes. 60% of *aub* mutant embryos and 90% of *armi* mutant embryos contained dispersed chromatin fragments that were often associated with small spindle-like microtubule bundles (Figure 1B, Table S2). The remaining embryos appeared to be progressing through cleavage divisions, and some cellularization and gastrulation stage embryos were observed. However, chromosome bridges/lagging chromosomes were present in 50% to 70% of the cleavage stage anaphase and early telophase figures (Figure 1B and Figure 2C).

Chromatin fragmentation could result from replication of broken chromosomes inherited from the female, or from post-fertilization fragmentation of the zygotic genome. To directly assay zygotic genome integrity, mutant females were mated to wild type males and dual-label FISH was used to monitor physically separate regions of the Y chromosome. In male embryos derived from wild type females, the two Y chromosome probes always co-segregated through anaphase and telophase (Figure 1C, 1D). Mutant embryos showing chromatin fragmentation, by contrast, contained chromatin clusters that did not label for either Y chromosome probe, or that labeled for only one of the two probes (Figure 1C). In mutant embryos that proceeded through cleavage stage mitotic cycles, the majority of segregating chromatids retained both Y chromosome markers, indicating that chromosome continuity had been maintained. Chromatids with only one of two markers were observed, however, indicating that breaks had separated regions on a Y chromosome arm from the centromere (Figure 1D). The axial patterning defects associated with piRNA mutations are suppressed by mutations in *mnk* [1], [2], but *mnk* did not suppress either the chromatin fragmentation or segregation defects linked to *aub* and *armi* (Table S2, Figure S3). Mutations in *aub* and *armi* thus destabilize the genome of the zygote and disrupt chromosome resolution during the cleavage divisions through processes that are independent of DNA damage signaling.

Mutations in the *armi* and *aub* genes disrupt piRNA production and transposon silencing, but have also been reported to inhibit homology dependent target cleavage by siRNAs [21], [22]. In addition, null mutations in *argonaute2 (ago2)*, which block siRNA based silencing, have been reported to disrupt mitosis during the syncytial blastoderm stage [23]. These observations raise the possibility that chromatin fragmentation and fusion in *aub* and *armi* mutants result from defects in the siRNA pathway. We therefore analyzed cleavage in embryos from females homozygous for null mutations in *ago2* and *dcr2*, which block siRNA production and silencing [24]. Consistent with previous studies, we find that embryos from *ago2* and *dcr2* mutant females are viable [23], [24]. However, we did not observe chromosome fragmentation or a statistically significant increase in anaphase bridge formation relative to wild type controls (Figure S4, Figure 2C). The *loquacious* (*loqs*) gene encodes a Dicer-1 binding protein required for miRNA production [25], and we find that embryos from *loqs* mutant females also proceed through normal cleavage stage divisions (Figure S4, Figure 2C). Chromosome segregation and maintenance of zygotic genome integrity during early embryogenesis thus appear to be independent of the siRNA and miRNA pathways, but require at least two components of the piRNA pathway.

### Telomere fusions in *aub* and *armi* embryos

In *S. pombe*, mutations in *ago1*, *dcr1* and *rdp1* disrupt kinetochore assembly and thus lead to lagging mitotic chromosomes due to defects in centromere movement to the spindle poles [26]. To determine if *Drosophila* piRNA mutations disrupt kinetochore assembly, we performed dual label FISH for centromeric dodeca-satellite sequences [27] and the telomere-specific transposon *HeT-A*. In *aub* and *armi* mutants, centromeric sequences segregated to the spindle poles in essentially every anaphase figure, but telomere specific sequences were consistently present at the chromatin bridges (Figure 2A). These observations indicate that *armi* and *aub* are not required for kinetochore assembly, but are needed for telomere resolution.

Telomeres are protected from recognition as DNA double strand breaks by the telomere-protection complex (TPC), and defects in telomere protection thus lead to covalent ligation of chromosome ends by the non-homologous end-joining (NHEJ) pathway [28], [29]. DNA Ligase IV is required for NHEJ, and *ligase IV* mutations suppress fusions that result from covalent joining of unprotected chromosome ends [28], [29]. To determine if chromosome fusions in *aub* and *armi* are due to NHEJ, we generated *ligIV;aub* and *ligIV;armi* double mutant females and analyzed chromosome segregation in the resulting embryos. In *aub* single mutant embryos, 50% of anaphase figures show bridges, but anaphase bridges are present in only 15% of *ligIV;aub* double mutants (Figure 2B, 2C). By contrast, the fraction of embryos showing chromosome fragmentation increases in *ligIV;aub* double mutants (Table S2). Chromosome fragmentation also increased in *ligIV;armi* mutant embryos, and as a result morphologically normal anaphase figures could not be observed (Table S2). These findings strongly suggest that lagging chromosomes result from covalent ligation of chromosome ends by the NHEJ pathway, while chromatin fragmentation results from DNA breaks that are repaired by NHEJ. Mutations in *armi* and *aub* lead to significant over-expression of transposable elements [8], [14], [30], including DNA elements that are mobilized by a “cut and paste” mechanism that directly produces double strand breaks [31]. In addition, NHEJ pathway has been implicated in repair of gapped retroviral integration intermediates [32]. Chromosome fragmentation may therefore result from transposon over-expression and mobilization, which induces breaks that overwhelm the NHEJ pathway. Telomere fusions, by contrast, appear to result from defects in telomere protection, which lead to chromosome end recognition by the NHEJ pathway.

### Assembly of the telomere protection complex

**Figure 3:**
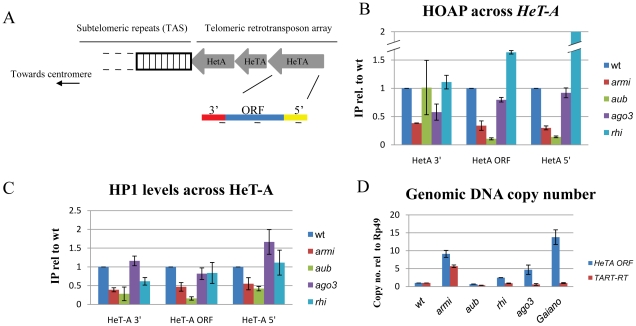
Mutations in *aub* and *armi* disrupt assembly of the telomere protection complex. A. Schematic showing transposon arrays at *Drosophila* telomeres. The *HeT-A* transposon 3′ and 5′-UTRs are in red and yellow respectively, and the ORF is in blue. B, C. Binding of the telomere protection complex proteins HOAP and HP1 to *HeT-A.* Chromatin Immunoprecipitation (ChIP) was used to recover bound DNA, and the percent of input chromatin precipitated was determined by qPCR. Fold change in binding relative to wild type is shown, and was calculated by dividing mutant by wild type (wt) values. D. Genomic copy number for *HeT-A* and *TART*. Copy number was determined by qPCR, using the single copy Rp49 gene as an internal standard. *Gaiano* is a wild-type stock previously shown to carry additional telomeric transposon repeats.

The *Drosophila* TPC includes HOAP and Modigliani (Moi), which may function only at chromosome ends, and HP1a and the MRN complex, which have additional roles in heterochromatic silencing and DNA repair [33], [34], [35], [36]. To directly assay for TPC recruitment, we used chromatin immunoprecipitation (ChIP) to measure HP1a and HOAP binding to the telomere specific transposon *HeT-A* (Figure 3B, 3C). In wild type ovaries, HOAP and HP1a bind to multiple regions of *HeT-*A (Figure 3B, 3C). In *armi* and *aub* mutants, by contrast, HOAP and HP1a binding to the *Het-A* 5′-UTR and ORF are significantly reduced (Figure 3B, 3C). The 5′end of *Het-A* is oriented toward the chromosome end, and is therefore likely to lie at the telomere. Ovarian tissue consists of germ cells with a surrounding layer of somatic cells, which complicates interpretation of these biochemical studies. However, ChIP on 0–3 hour old embryos from *aub* and *mnk,aub* mutant females revealed significant reduction in HOAP binding at the *HeT-A* 5′-UTR (Figure S5). The *aub* and *armi* genes thus appear to be required for TPC recruitment, consistent with ligation of chromosome ends in mutant embryos.

To determine if other piRNA pathway mutations disrupt telomere protection, we analyzed the cleavage stage embryonic divisions in *ago3* and *rhi* mutants. The *ago3* locus encodes a PIWI clade protein that primarily binds sense strand piRNAs, and *rhi* encodes a rapidly evolving HP1 homologue required for production of precursor RNAs from a subset of piRNA clusters [14], [30]. Essentially all of the *rhi* and *ago3* mutant embryos showed chromatin fragmentation, as observed in the majority of *aub* and *armi* mutants (Figure S6). We therefore biochemically assayed for TPC assembly in ovarian chromatin using ChIP for HOAP and HP1a. Surprisingly, neither *ago3* nor *rhi* mutations disrupt HOAP or HP1a binding to *Het-A*, and *rhi* mutants show greater than wild type levels of HOAP binding to *Het-A* (Figure 3B, 3C). By contrast, these *rhi* alleles reduce total piRNA production by 10 fold [14]. The *ago3* mutations appear to be null, and the *rhi* mutations are strong hypomorphc alleles. Assembly of the TPC in the *ago3* and *rhi* mutants is therefore unlikely to be mediated by residual protein. Instead, these findings strongly suggest that *aub* and *armi* have a function in telomere protection that is not shared by *ago3* or *rhi*.

In *Drosophila*, chromosome breaks can be converted to stable telomeres [37], called terminal deletions, which accumulate additional copies of the telomeric elements *HeT-A* and *TART*. When terminal deletions are passaged in animals heterozygous for *aub* or the piRNA pathway gene *spnE*, the number of terminal *TART* repeats increase[38]. The defects in TPC assembly in *aub* and *armi* could therefore be triggered by increased *HeT-A* and *TART* copy number, which could titrate TPC components. We therefore assayed telomeric transposon copy number in *aub* and *armi* mutants, which show defects in TPC assembly, and in *rhi* and *ago3* mutants, which do not. We also assayed telomeric transposon copy number and mitotic chromosome segregation in a wild-type variant, *Gaiano*, that has been reported to carry additional *HeT-A* repeats [39]. Consistent with previous reports, we find that *Gaiano* has 10 to 15 fold more *HeT-A* copies than *OregonR* controls (Figure 3D). Despite the increase in telomere length, this stock is viable and fertile, and we did not observe telomere fusions or lagging chromosomes during the cleavage stage embryonic divisions (Figure S6). In addition, we found that *aub* mutants that show defects in TPC assembly do not accumulate additional copies of *HeT-A* or *TART*, while *rhi* and *ago3* mutants that are wild type for TPC binding show an increase in telomere-specific transposon copy number (Figure 3D). Assembly of the TPC is therefore independent of telomere specific transposon copy number (Figure S6).

### Aub and Armi are required for production of a subpopulation of 19–22 nt piRNAs

**Figure 4:**
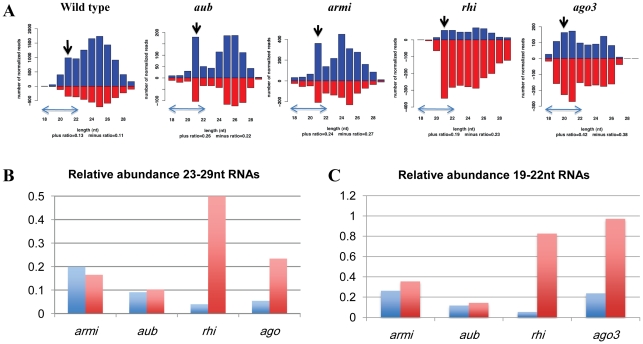
piRNAs linked to a 4^th^ chromosome cluster containing telomeric transposon fragments. A. Length histograms showing plus genomic strand (blue) and minus genomic strand (red) mapping piRNAs in wt, *armi*, *aub*, *rhi* and *ago3* mutants. The relative abundance is normalized to sequencing depth and is plotted on the y-axis. Note that sense strand of the transposon fragments in this cluster are on the minus genomic strand, and that the scales differ. Preferential loss of shorter piRNAs from *aub* and *armi* leads to a prominent endo-siRNA peak at 21 nt (marked by a black arrow). B. Abundance of longer (23–29 nt) plus strand (blue) and minus strand (red) piRNAs in the indicated mutants relative to their respective wild-type controls. All four mutations reduce plus strand piRNAs, which are anti-sense to the telomeric transposons. C. 19–22 nt genomic plus and minus strand piRNAs in the indicated mutants. All four mutations reduce plus strand RNAs. However, minus strand species are retained at near wild type levels in both *rhi* and *ago3* mutants. For panels B and C, bars show normalized reads in mutants divided by normalized reads in wild-type controls.

piRNAs are proposed to direct PIWI clade proteins to targets through sequence specific interactions. Our observations raised the possibility that *armi* and *aub* promote production of piRNAs that direct the telomere protection complex to transposons that make up chromsome ends. We therefore analyzed published small RNA deep sequencing data[14], [15], [30] for species derived from a fourth chromosome cluster, defined by a high density of uniquely mapping piRNAs, containing multiple repeats of the telomeric transposons [40]. Our bioinformatic analysis showed that 70–80% of telomere specific piRNAs match this cluster (Figure 4, Table S3). Figure 4 shows length histograms for small RNAs from wt, *rhi*, *ago3*, *aub* and *armi* mutant ovaries that map to this cluster. Data are normalized to sequencing depth, and small RNAs mapping to the plus genomic strand are represented in blue and RNAs mapping to the minus strand are in red. Significantly, *aub* and *armi* mutations lead to a preferential loss of shorter piRNAs mapping to the minus genomic strand (Figure 4B, 4C). Loss of these shorter RNAs highlights the peak at 21 nt, which is retained in all of the mutants and likely represent endogenous siRNAs (Figure 4A, black arrow). The telomeric elements (*HeT-A* and *TART*) are almost exclusively on the minus genomic strand in this cluster, and the RNAs that are lost in *aub* and *armi* thus correspond to the sense strand of the target elements. Ovaries mutant for *ago3* and *rhi*, by contrast, retain these shorter sense strand RNAs.

We quantified the relative abundance of typical 23–29nt long piRNAs and the shorter 19–22nt species, excluding the 21nt endo-siRNA peak. All four mutations significantly reduce 23 to 29 nt piRNAs, although *rhi* mutants retain approximately 50% of wild type minus strand species. Loss of these piRNAs is consistent with over-expression of transposons matching this cluster in all four mutants (Figure S8). By contrast, the shorter minus strand RNAs are reduced by 3 to 10 fold in *armi* and *aub*, but are expressed at 80% to 95% of wild type levels in *ago3* and *rhi* (Figure 4B, 4C). In addition, short piRNA species from the telomeric cluster co-immunoprecipitate with Piwi protein [15], [30], which localizes to the nucleus and is a likely effector of chromatin functions for the piRNA pathway (Figure S7). Binding of this subpopulation of piRNAs by Piwi is retained in *ago3* mutants, which assemble the TPC, but significantly reduced in *armi* mutants, which block assembly of the TPC (Figure S7).

Taken together, these observations suggest that the piRNA pathway has two genetically distinct functions during oogenesis and early embryogenesis. The pathway prevents DNA damage during oogenesis and maintains the integrity of the zygotic genome during the embryonic cleavage divisions, which likely reflects the established role for piRNAs in transposon silencing [2], [8], [14], [30]. This function requires *aub*, *armi*, *rhi* and *ago3*, which are also required for wild type piRNA production. In addition, our studies reveal a novel function for the piRNA genes *aub* and *armi* in telomere protection, whch may be mediated by a novel class of short RNAs that bind to Piwi. Consistent with this hypothesis, it has been reported that germline clones of *piwi* null alleles do not significantly disrupt oogenesis, but lead to maternal effect embryonic lethality and severe chromosome segregation defects during the cleavage divisions [41]. A subpopulation of Piwi-bound piRNAs may therefore direct assembly of the TPC.

## Materials and Methods

### Fly stocks

Flies were reared at 25°C on standard corn meal medium. *OregonR* and *w1118* were used as controls. Stocks carrying the following alleles were obtained from the Bloomington Stock Center: *ago2^51B^*, *ago2^Df^*, *aub^HN2^*, *aub^QC42^*, *dcr2^L811fsX^*, *mnk^P6^*, *ligIV^5^*, *rhi^02086^* and *rhi^KG00910^* . *ago2^51B^* is an imprecise P-element induced deletion of the first two exons of *ago2* locus. *aub^HN2^* and *aub^QC42^*are both EMS-induced point mutations [42], [43]. *dcr2^L811fsX^* is an EMS-induced loss-of-function allele described in [24]. *rhi^02086^* and *rhi^KG00910^* are both P-element insertion alleles, which act as strong hypomorphs [44]. Both *armi^1^*and *armi^72.1^*alleles are strong hypomorphic alleles which produce *armi* transcript at low levels [4]. *mnk^P6^,aub^HN2^* and *mnk^P6^,aub^QC42^* [2] recombinants were generated using standard genetic procedures. The *loqs^f00791^* and *loqs^KO^* alleles were from Bloomington and Dennis McKearin [25], respectively. Stocks carrying *ago3^4931^*and *ago3^3658^*, which are loss-of-function alleles with premature stop codons [30], were obtained from the Zamore lab (University of Massachusetts Medical School).

### Immunostaining and fluorescence in situ hybridization

0–30-min-old or 0–3-hr-old embryos were fixed in methanol and immunostained for α-tubulin (Dm1α, Sigma Chemical Co., 1∶300) and 0.2 µM TOTO-3 (Molecular Probes) using standard procedures [45]. For staining of egg chambers, the ovaries were dissected in Robb's medium and fixed in 4% formaldehyde as described [2]. γ-H2Av antibody was generously provided by Kim McKim (Rutgers) and was used at 1∶500 dilution. The dodeca-satellite probe for the fluorescent in situ hybridization was made by 3′ end labeling using terminal deoxynucleotidyl transferase (Roche), followed by direct fluorophore conjugation using ARES DNA labeling kit as described by the manufacturer (Molecular Probes). The dodeca satellite sequence from the pBK6E218 plasmid was amplified using T3 and T7 primers [27]. The telomeric probe was made by indirect substitution of DIG-dUTP using the PCR DIG probe synthesis kit (Roche). The sequence was amplified from genomic DNA using the following primers- telF- 5′-GACAATGCACGACAGAGGAA-3′ and telR- 5′-GTCTTTTTGGGTTTGCGGTA-3′. The Y-chromosome satellites (AATAC)n and (AATAAAC)n were purchased as oligos with direct conjugation of FAM and Cy-3 fluorophores at the 3′end (IDT). Hybridization was performed as described previously [46]. Fluorescently labeled samples were imaged using a Leica TCS-SP inverted scanning confocal microscope or a Nikon TE-2000E2 inverted microscope and captured using Metamorph software (Universal Imaging). All images were processed using Image J (Rasband, W.S., ImageJ, U.S. National Institutes of Health, Bethesda, Maryland, USA, http://rsb.info.nih.gov/ij/, 1997–2006) and Adobe Photoshop.

### Chromatin bridges quantification

To quantify chromatin bridges, the ratio of anaphase/telophase (A/T) bridges to total A/T figures was calculated for 10 to 30 embryos. The mean bridge frequency was determined by designating each embryo as an independent experiment, and the standard error was determined using an Anova test. Two-tailed t-tests were also used to compare specific data sets, using α = 0.05. P-values are noted on the graphs.

### Chromatin Immunoprecipitation and quantitative PCR (qPCR)

Whole ovaries were dissected from 2–5-day old flies and fixed using 1.8% formaldehyde for 10 minutes at room temperature. For ChIP using embryos, 0–3 hr old embryos were collected and fixed using 1.8% formaldehyde for 20 minutes at room temperature. The ChIP assay was performed as per manufacturer's instructions (Invitrogen) and as previously described with some modifications [14]. Immunoprecipitation was done using HOAP polyclonal serum previously described [14] or the monoclonal HP1 antibody (Developmental Studies Hybridoma Bank, IA). The purified DNA was subjected to qPCR using Applied Biosystems 7500 system, and data was analyzed by calculating the % of immunoprecipitated DNA compared to the input DNA sample. All ChIPs were performed at least twice and the data presented is an average of two different biological replicates with technical triplicates for each of them. The data was plotted with error bars representing standard deviations for individual samples. The difference between primer efficiencies was calculated by preparing standard curves and was taken into consideration while calculating % IP values. The primer sequences are available upon request.

### Sequence extraction and annotation

For each sequence read, the first occurrence of the 6-mer perfectly matching the 5′-end of the 3′-linker was identified. Sequences without a match were discarded. The extracted inserts for sequences that contained the 3′-linker were then mapped to the female *Drosophila melanogaster* genome (Release R5.5, excluding chromosome YHet). Inserts that matched fully to a genomic sequence were collected using Bowtie (Langmead et al., 2009) and the corresponding genomic coordinates were determined for downstream functional analysis. Sequences corresponding to pre-miRNAs or non-coding RNAs (ncRNAs) were identified and removed. For analyis of the telomeric cluster, small RNA length distributions were determined for reads that mapping to chr4:1280000–1350999, normalizing for sequencing depth (genome mapping reads excluding ncRNAs).

## Supporting Information

Figure S1 DNA breaks in the piRNA mutants disappear by the end of oogenesis. Immunostaining of ovaries from *OregonR* control, *aub* and *armi* mutants for γ-H2Av(green) and DNA (blue) during stage 3, 5 and 8 of oogenesis showing the disappearance of the γ-H2Av signal by late stages. The oocyte is marked by a solid trace path.
(2.46 MB TIF)

Figure S2 Mature oocytes in piRNA mutants show compact chromatin mass. Overview of stage 14 oocytes in *OregonR*, *armi*, and *aub* females stained for DNA.
(1.81 MB TIF)

Figure S3 DNA bridges in piRNA mutants are independent of Chk2 activation Immunostaining of DNA (blue) and microtubules (green) in embryos from *mnk*, *mnk armi* and *mnk aub* showing chromatin bridges during syncytial mitotic divisions.
(2.47 MB TIF)

Figure S4 Chromosome segregation in RNAi and miRNA mutants. Immunostaining of DNA (blue) and microtubules (green) in embryos from *ago2*, *dcr2* and *loquacious loqs* showing normal chromosome segregation during syncytial mitotic divisions.
(1.25 MB TIF)

Figure S5 HOAP recruitment defect in early embryos ChIP-qPCR analysis of HOAP antibody from 0–3-hr old embryos in wt, *aub* and *mnk aub* across telomeric regions.
(0.13 MB TIF)

Figure S6 Cleavage stage embryos mutant for *rhi* and *ago3*. *Gaiano* is a wild type strain carrying additional telomeric repeats. The *rhi* and *ago3* mutations lead to chromosome fragmentation. Mitosis is normal in *Gaiano* embyro. DNA is in blue and microtubules in green.
(0.82 MB TIF)

Figure S7 Telomeric cluster piRNAs bound to Piwi in wild type, *ago3*, and *armi* mutant ovaries. Length histograms are shown in B and piRNA distributions across the cluster are shown in B.
(3.00 MB TIF)

Figure S8 Expression of telomeric elements in piRNA mutants. Genome browser views of expression from the forth chromosome telomeric array are shown. All four of the indicated mutations lead to over-expression of these elements.
(3.00 MB TIF)

Table S1 4th chromosome morphology in stage 13 oocytes.
(0.03 MB DOC)

Table S2 Percentage of embryos from different genotypes showing chromatin fragmentation.
(0.03 MB DOC)

Table S3 Contribution of piRNAs against telomeric transposons from the 4th chromosome cluster.
(0.03 MB DOC)
