# Supplementary figures and images for: Distinct Functions for the Drosophila piRNA Pathway in Genome Maintenance and Telomere Protection
Source: PLoS Genet. 2010 Dec 16;6(12):e1001246. doi: 10.1371/journal.pgen.1001246 (PMC3003142; doi:10.1371/journal.pgen.1001246)

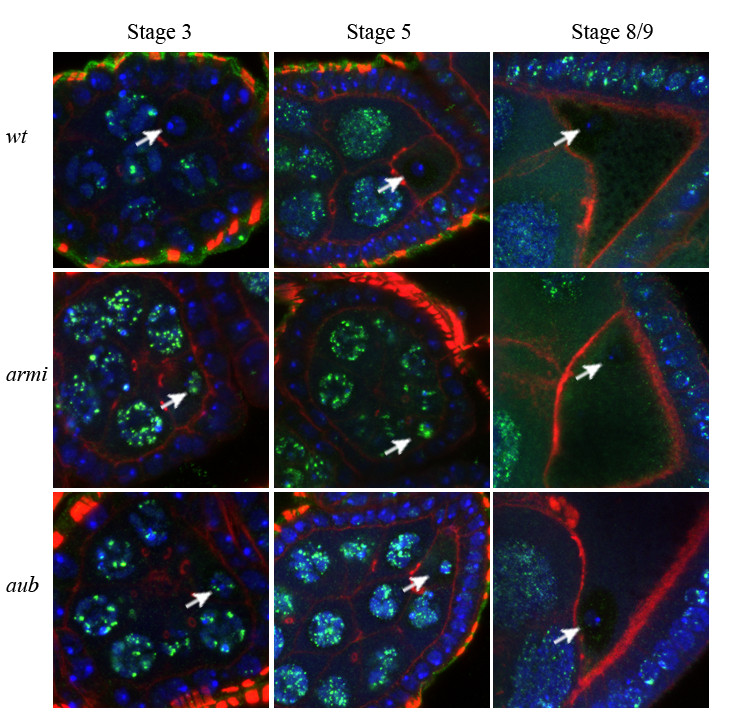

Supplement: Figure S1 — DNA breaks in the piRNA mutants disappear by the end of oogenesis. Immunostaining of ovaries from OregonR control, aub and armi mutants for γ-H2Av(green) and DNA (blue) during stage 3, 5 and 8 of oogenesis showing the disappearance of the γ-H2Av signal by late stages. The oocyte is marked by a solid trace path. (2.46 MB TIF) [file pgen.1001246.s001.tif]

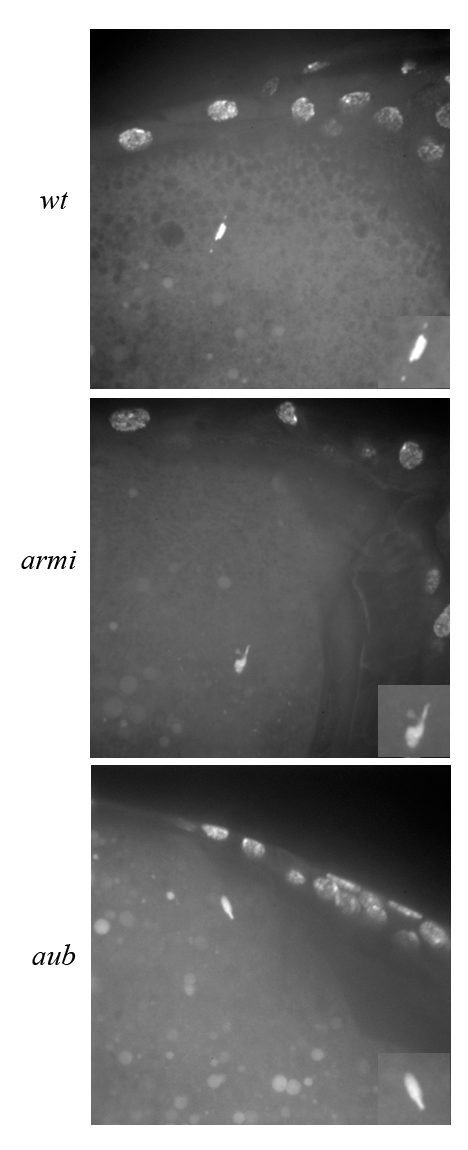

Supplement: Figure S2 — Mature oocytes in piRNA mutants show compact chromatin mass. Overview of stage 14 oocytes in OregonR, armi, and aub females stained for DNA. (1.81 MB TIF) [file pgen.1001246.s002.tif]

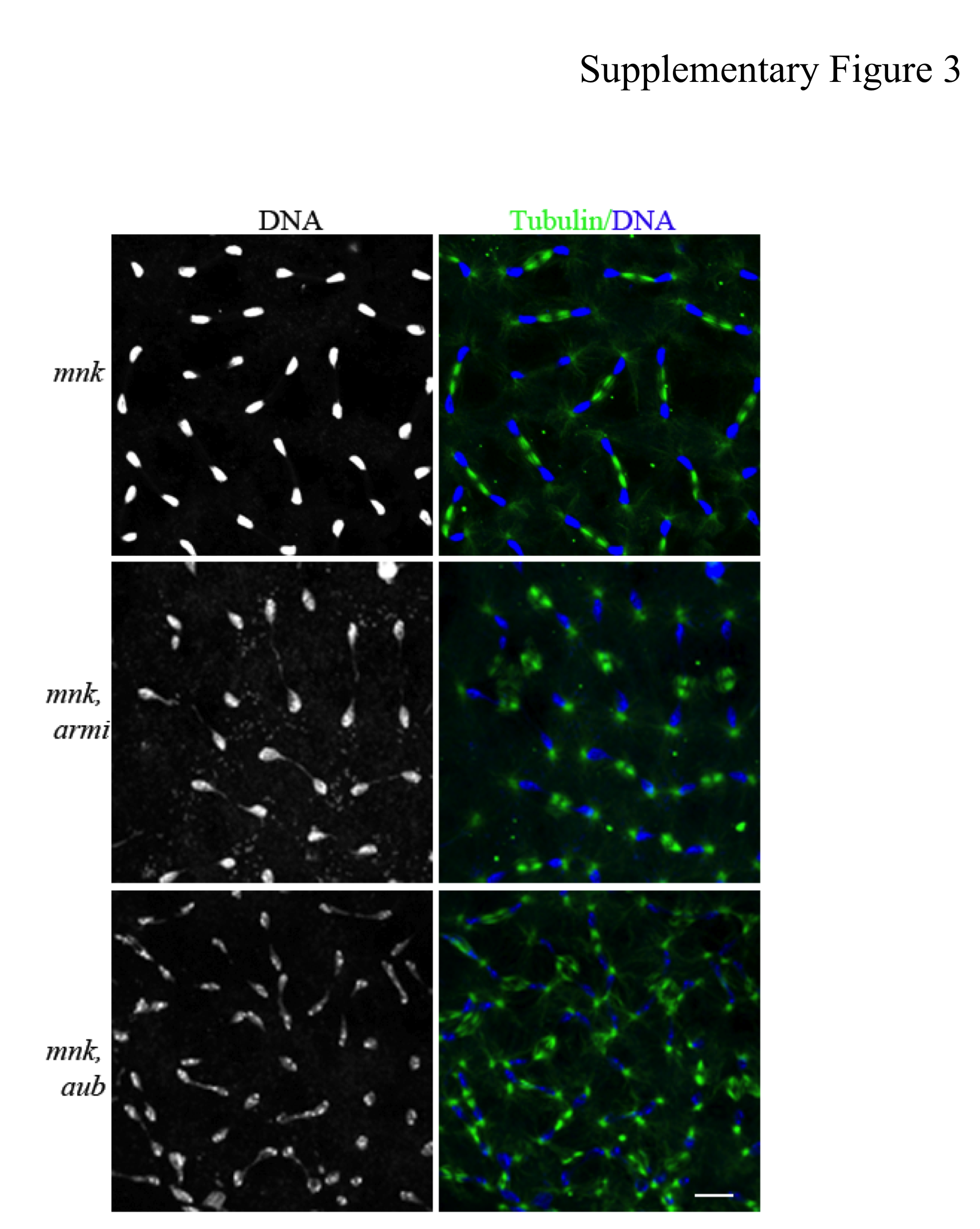

Supplement: Figure S3 — DNA bridges in piRNA mutants are independent of Chk2 activation Immunostaining of DNA (blue) and microtubules (green) in embryos from mnk, mnk armi and mnk aub showing chromatin bridges during syncytial mitotic divisions. (2.47 MB TIF) [file pgen.1001246.s003.tif]

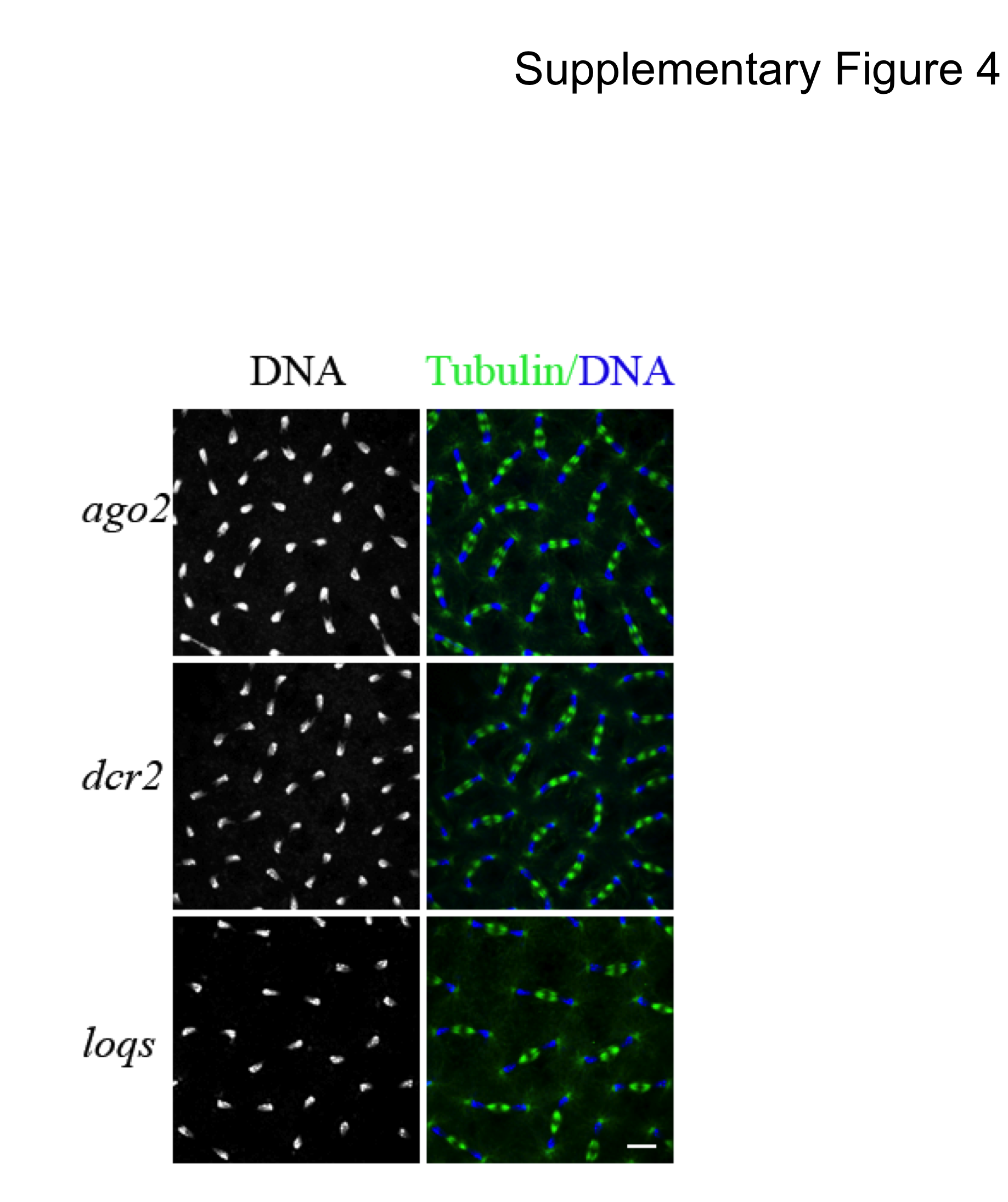

Supplement: Figure S4 — Chromosome segregation in RNAi and miRNA mutants. Immunostaining of DNA (blue) and microtubules (green) in embryos from ago2, dcr2 and loquacious loqs showing normal chromosome segregation during syncytial mitotic divisions. (1.25 MB TIF) [file pgen.1001246.s004.tif]

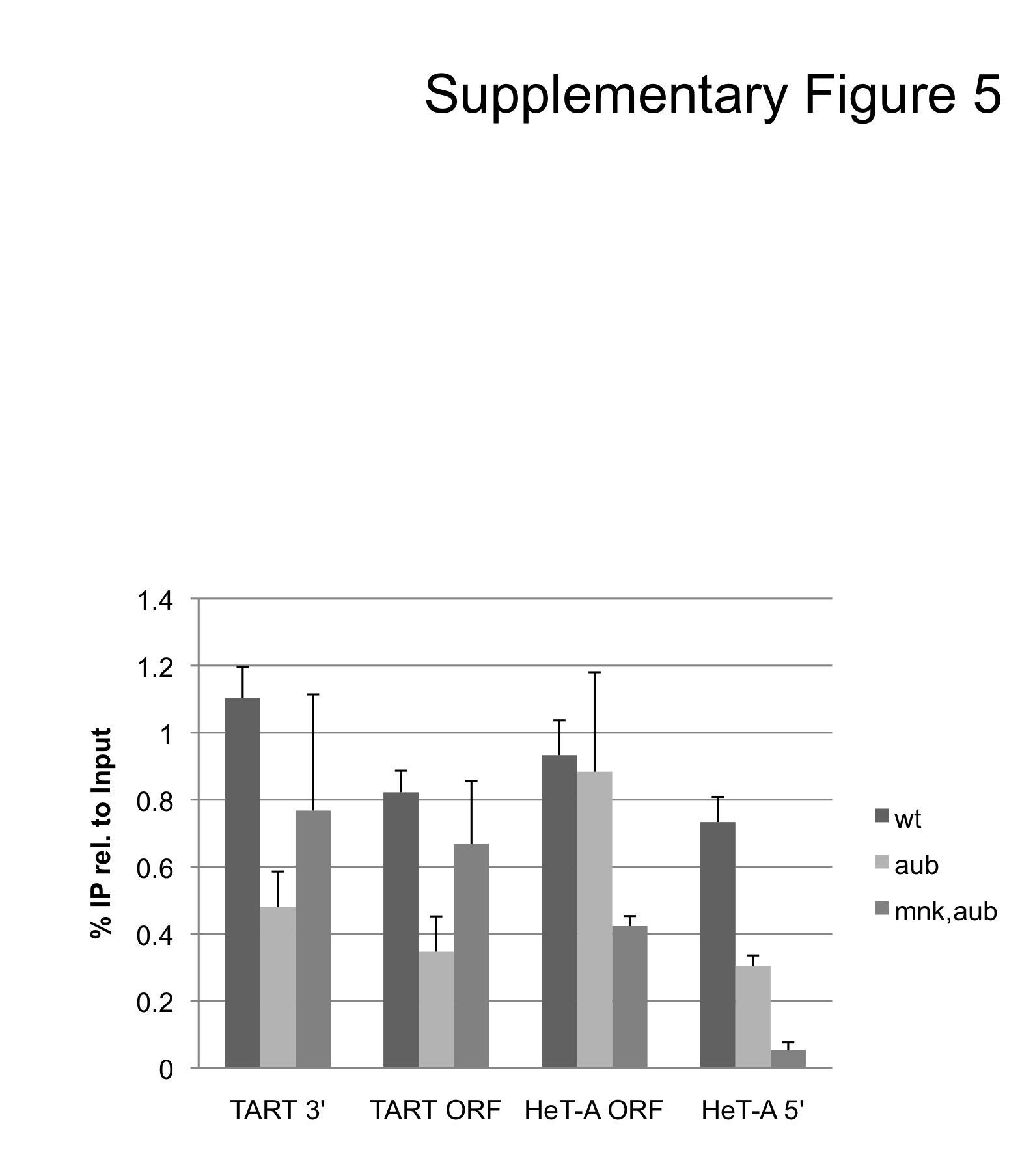

Supplement: Figure S5 — HOAP recruitment defect in early embryos ChIP-qPCR analysis of HOAP antibody from 0–3-hr old embryos in wt, aub and mnk aub across telomeric regions. (0.13 MB TIF) [file pgen.1001246.s005.tif]

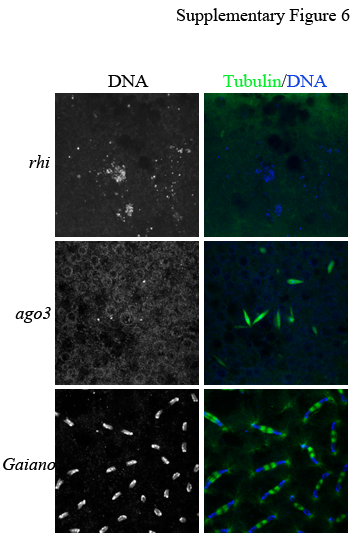

Supplement: Figure S6 — Cleavage stage embryos mutant for rhi and ago3. Gaiano is a wild type strain carrying additional telomeric repeats. The rhi and ago3 mutations lead to chromosome fragmentation. Mitosis is normal in Gaiano embyro. DNA is in blue and microtubules in green. (0.82 MB TIF) [file pgen.1001246.s006.tif]

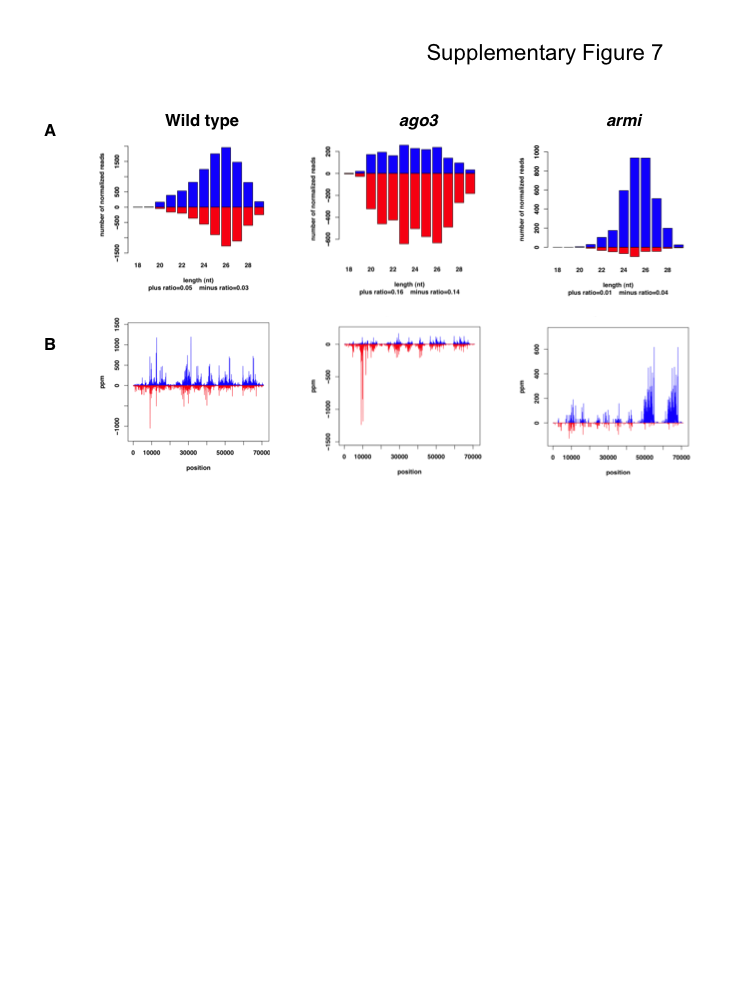

Supplement: Figure S7 — Telomeric cluster piRNAs bound to Piwi in wild type, ago3, and armi mutant ovaries. Length histograms are shown in B and piRNA distributions across the cluster are shown in B. (3.00 MB TIF) [file pgen.1001246.s007.tif]

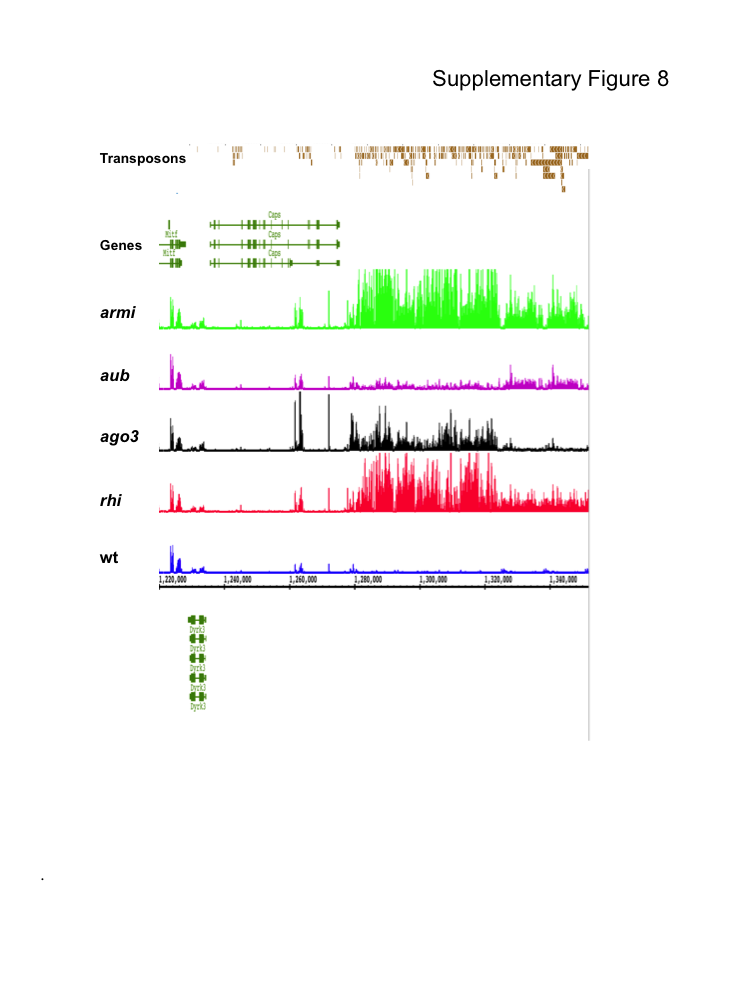

Supplement: Figure S8 — Expression of telomeric elements in piRNA mutants. Genome browser views of expression from the forth chromosome telomeric array are shown. All four of the indicated mutations lead to over-expression of these elements. (3.00 MB TIF) [file pgen.1001246.s008.tif]
