# Supplementary material for: Distinct Functions for the Drosophila piRNA Pathway in Genome Maintenance and Telomere Protection
Source: PLoS Genet. 2010 Dec 16;6(12):e1001246. doi: 10.1371/journal.pgen.1001246 (PMC3003142; doi:10.1371/journal.pgen.1001246)
Supplement: Table S1 — 4th chromosome morphology in stage 13 oocytes. (0.03 MB DOC) [file pgen.1001246.s009.doc]

Supplementary Table 1

| **Genotype** | **Percentage of Stage 13 oocytes showing separate 4th chromosomes** | **Number of oocytes scored** |
| --- | --- | --- |
| *OregonR* | 78.57 | 28 |
| *armi^1/72.1^* | 17.65 | 17 |
| *aub^HN/QC^* | 11.11 | 18 |
