# Supplementary material for: Distinct Functions for the Drosophila piRNA Pathway in Genome Maintenance and Telomere Protection
Source: PLoS Genet. 2010 Dec 16;6(12):e1001246. doi: 10.1371/journal.pgen.1001246 (PMC3003142; doi:10.1371/journal.pgen.1001246)
Supplement: Table S2 — Percentage of embryos from different genotypes showing chromatin fragmentation. (0.03 MB DOC) [file pgen.1001246.s010.doc]

Supplementary Table 2

| **Genotype** | **Fragmented** | **Cycling** | **Mixed** |
| --- | --- | --- | --- |
| *aub^HN2/QC42^* | 60 | 38 | 2 |
| *mnk^p6^,aub^HN2^/mnk^p6^,aub^QC42^* | 40 | 59 | 1 |
| *ligIV^5^/ ligIV^5^;aub^HN2^/aub^QC42^* | 73 | 11 | 16 |
| *armi^1^/armi^72.1^* | 88 | 10 | 2 |
| *mnk^p6^/mnk^p6^;armi^1^/armi^72.1^* | 77 | 13 | 11 |
| *ligIV^5^/ligIV^5^;armi^1^/armi^72.1^* | 92 | 2 | 7 |
