# Supplementary material for: Distinct Functions for the Drosophila piRNA Pathway in Genome Maintenance and Telomere Protection
Source: PLoS Genet. 2010 Dec 16;6(12):e1001246. doi: 10.1371/journal.pgen.1001246 (PMC3003142; doi:10.1371/journal.pgen.1001246)
Supplement: Table S3 — Contribution of piRNAs against telomeric transposons from the 4th chromosome cluster. (0.03 MB DOC) [file pgen.1001246.s011.doc]

Supplementary Table 3

| **Telomeric element** | **piRNAs unique to chr. 4 telomeric cluster** | **piRNAs unique to other chromosomes** | **Total piRNAs shared** | **Fraction coming from chr 4** | |
| --- | --- | --- | --- | --- | --- |
| *HeT A* | 2907 | 3108 | 9172 | 0.79 |  |
| *TART-A* | 2355 | 3274 | 6506 | 0.73 |  |
| *TAHRE* | 1631 | 3167 | 6507 | 0.71 |  |
